# Supplementary material for: Pyruvate Dehydrogenase Kinase 1 inhibition mediated oxidative phosphorylation enhancement in cartilage promotes osteoarthritis progression
Source: BMC Musculoskelet Disord. 2023 Jul 20;24:597. doi: 10.1186/s12891-023-06585-6 (PMC10357736; doi:10.1186/s12891-023-06585-6)
Supplement: Supplementary file 1 — Additional file 1. [file 12891_2023_6585_MOESM1_ESM.zip › KEGG.Gsea.1653622432464/KEGG_VASCULAR_SMOOTH_MUSCLE_CONTRACTION.html]

Details for gene set KEGG\_VASCULAR\_SMOOTH\_MUSCLE\_CONTRACTION[GSEA]

|  || Dataset | 55457\_collapsed\_to\_symbols.cls.cls #OA\_versus\_normal.cls.cls #OA\_versus\_normal\_repos |
| Phenotype | cls.cls#OA\_versus\_normal\_repos |
| Upregulated in class | normal |
| GeneSet | KEGG\_VASCULAR\_SMOOTH\_MUSCLE\_CONTRACTION |
| Enrichment Score (ES) | -0.19712913 |
| Normalized Enrichment Score (NES) | -0.8541372 |
| Nominal p-value | 0.75384617 |
| FDR q-value | 0.88352215 |
| FWER p-Value | 1.0 |
Table: GSEA Results Summary

  

Fig 1: Enrichment plot: KEGG\_VASCULAR\_SMOOTH\_MUSCLE\_CONTRACTION      
 Profile of the Running ES Score & Positions of GeneSet Members on the Rank Ordered List

  

| SYMBOL | TITLE | RANK IN GENE LIST | RANK METRIC SCORE | RUNNING ES | CORE ENRICHMENT || 1 | KCNMB1 | potassium calcium-activated channel subfamily M regulatory beta subunit 1 [Source:HGNC Symbol;Acc:HGNC:6285] | 192 | 0.827 | 0.0135 | No |
| 2 | PLA2G12A | phospholipase A2 group XIIA [Source:HGNC Symbol;Acc:HGNC:18554] | 600 | 0.623 | 0.0032 | No |
| 3 | PPP1CA | protein phosphatase 1 catalytic subunit alpha [Source:HGNC Symbol;Acc:HGNC:9281] | 603 | 0.622 | 0.0245 | No |
| 4 | PPP1CC | protein phosphatase 1 catalytic subunit gamma [Source:HGNC Symbol;Acc:HGNC:9283] | 744 | 0.583 | 0.0337 | No |
| 5 | MAPK3 | mitogen-activated protein kinase 3 [Source:HGNC Symbol;Acc:HGNC:6877] | 757 | 0.579 | 0.0527 | No |
| 6 | PRKACB | protein kinase cAMP-activated catalytic subunit beta [Source:HGNC Symbol;Acc:HGNC:9381] | 771 | 0.576 | 0.0715 | No |
| 7 | MYL6B | myosin light chain 6B [Source:HGNC Symbol;Acc:HGNC:29823] | 815 | 0.565 | 0.0876 | No |
| 8 | AVPR1B | arginine vasopressin receptor 1B [Source:HGNC Symbol;Acc:HGNC:896] | 865 | 0.552 | 0.1028 | No |
| 9 | CALM3 | calmodulin 3 [Source:HGNC Symbol;Acc:HGNC:1449] | 1039 | 0.510 | 0.1069 | No |
| 10 | CALML5 | calmodulin like 5 [Source:HGNC Symbol;Acc:HGNC:18180] | 1261 | 0.465 | 0.1057 | No |
| 11 | KCNMB4 | potassium calcium-activated channel subfamily M regulatory beta subunit 4 [Source:HGNC Symbol;Acc:HGNC:6289] | 1361 | 0.449 | 0.1134 | No |
| 12 | ADCY7 | adenylate cyclase 7 [Source:HGNC Symbol;Acc:HGNC:238] | 1408 | 0.442 | 0.1251 | No |
| 13 | KCNMA1 | potassium calcium-activated channel subfamily M alpha 1 [Source:HGNC Symbol;Acc:HGNC:6284] | 1573 | 0.418 | 0.1266 | No |
| 14 | ADCY1 | adenylate cyclase 1 [Source:HGNC Symbol;Acc:HGNC:232] | 1589 | 0.415 | 0.1398 | No |
| 15 | PTGIR | prostaglandin I2 receptor [Source:HGNC Symbol;Acc:HGNC:9602] | 2055 | 0.356 | 0.1157 | No |
| 16 | PLCB4 | phospholipase C beta 4 [Source:HGNC Symbol;Acc:HGNC:9059] | 2217 | 0.337 | 0.1147 | No |
| 17 | PRKCD | protein kinase C delta [Source:HGNC Symbol;Acc:HGNC:9399] | 2237 | 0.335 | 0.1248 | No |
| 18 | PLA2G4A | phospholipase A2 group IVA [Source:HGNC Symbol;Acc:HGNC:9035] | 2656 | 0.284 | 0.1020 | No |
| 19 | CACNA1F | calcium voltage-gated channel subunit alpha1 F [Source:HGNC Symbol;Acc:HGNC:1393] | 2726 | 0.278 | 0.1061 | No |
| 20 | PLA2G10 | phospholipase A2 group X [Source:HGNC Symbol;Acc:HGNC:9029] | 3315 | 0.220 | 0.0678 | No |
| 21 | KCNMB2 | potassium calcium-activated channel subfamily M regulatory beta subunit 2 [Source:HGNC Symbol;Acc:HGNC:6286] | 3637 | 0.191 | 0.0493 | No |
| 22 | PLA2G3 | phospholipase A2 group III [Source:HGNC Symbol;Acc:HGNC:17934] | 3648 | 0.190 | 0.0550 | No |
| 23 | PRKCQ | protein kinase C theta [Source:HGNC Symbol;Acc:HGNC:9410] | 3658 | 0.189 | 0.0609 | No |
| 24 | PLCB1 | phospholipase C beta 1 [Source:HGNC Symbol;Acc:HGNC:15917] | 3724 | 0.183 | 0.0621 | No |
| 25 | PRKCA | protein kinase C alpha [Source:HGNC Symbol;Acc:HGNC:9393] | 3728 | 0.183 | 0.0682 | No |
| 26 | ADORA2A | adenosine A2a receptor [Source:HGNC Symbol;Acc:HGNC:263] | 3870 | 0.168 | 0.0630 | No |
| 27 | RAMP3 | receptor activity modifying protein 3 [Source:HGNC Symbol;Acc:HGNC:9845] | 3948 | 0.160 | 0.0625 | No |
| 28 | GUCY1B1 | guanylate cyclase 1 soluble subunit beta 1 [Source:HGNC Symbol;Acc:HGNC:4687] | 3962 | 0.159 | 0.0670 | No |
| 29 | CACNA1S | calcium voltage-gated channel subunit alpha1 S [Source:HGNC Symbol;Acc:HGNC:1397] | 4031 | 0.153 | 0.0669 | No |
| 30 | RAMP1 | receptor activity modifying protein 1 [Source:HGNC Symbol;Acc:HGNC:9843] | 4201 | 0.138 | 0.0585 | No |
| 31 | ITPR2 | "inositol 1,4,5-trisphosphate receptor type 2 [Source:HGNC Symbol;Acc:HGNC:6181]" | 4222 | 0.135 | 0.0616 | No |
| 32 | ACTA2 | "actin alpha 2, smooth muscle [Source:HGNC Symbol;Acc:HGNC:130]" | 4490 | 0.114 | 0.0446 | No |
| 33 | ADRA1A | adrenoceptor alpha 1A [Source:HGNC Symbol;Acc:HGNC:277] | 4556 | 0.109 | 0.0433 | No |
| 34 | EDNRA | endothelin receptor type A [Source:HGNC Symbol;Acc:HGNC:3179] | 4663 | 0.099 | 0.0384 | No |
| 35 | MYL9 | myosin light chain 9 [Source:HGNC Symbol;Acc:HGNC:15754] | 4939 | 0.077 | 0.0196 | No |
| 36 | ACTG2 | "actin gamma 2, smooth muscle [Source:HGNC Symbol;Acc:HGNC:145]" | 4940 | 0.077 | 0.0222 | No |
| 37 | PLA2G2D | phospholipase A2 group IID [Source:HGNC Symbol;Acc:HGNC:9033] | 5056 | 0.069 | 0.0156 | No |
| 38 | PPP1R12B | protein phosphatase 1 regulatory subunit 12B [Source:HGNC Symbol;Acc:HGNC:7619] | 5058 | 0.068 | 0.0179 | No |
| 39 | MYLK | myosin light chain kinase [Source:HGNC Symbol;Acc:HGNC:7590] | 5079 | 0.066 | 0.0186 | No |
| 40 | ADCY9 | adenylate cyclase 9 [Source:HGNC Symbol;Acc:HGNC:240] | 5191 | 0.057 | 0.0119 | No |
| 41 | MYH11 | myosin heavy chain 11 [Source:HGNC Symbol;Acc:HGNC:7569] | 5211 | 0.056 | 0.0124 | No |
| 42 | ADRA1B | adrenoceptor alpha 1B [Source:HGNC Symbol;Acc:HGNC:278] | 5275 | 0.050 | 0.0092 | No |
| 43 | MYLK3 | myosin light chain kinase 3 [Source:HGNC Symbol;Acc:HGNC:29826] | 5294 | 0.048 | 0.0094 | No |
| 44 | PRKCE | protein kinase C epsilon [Source:HGNC Symbol;Acc:HGNC:9401] | 5926 | 0.000 | -0.0399 | No |
| 45 | AGTR1 | angiotensin II receptor type 1 [Source:HGNC Symbol;Acc:HGNC:336] | 6122 | -0.014 | -0.0546 | No |
| 46 | RAF1 | "Raf-1 proto-oncogene, serine/threonine kinase [Source:HGNC Symbol;Acc:HGNC:9829]" | 6187 | -0.019 | -0.0590 | No |
| 47 | CALML3 | calmodulin like 3 [Source:HGNC Symbol;Acc:HGNC:1452] | 6348 | -0.032 | -0.0704 | No |
| 48 | CACNA1C | calcium voltage-gated channel subunit alpha1 C [Source:HGNC Symbol;Acc:HGNC:1390] | 6367 | -0.033 | -0.0706 | No |
| 49 | PRKCB | protein kinase C beta [Source:HGNC Symbol;Acc:HGNC:9395] | 6453 | -0.039 | -0.0760 | No |
| 50 | ARHGEF12 | Rho guanine nucleotide exchange factor 12 [Source:HGNC Symbol;Acc:HGNC:14193] | 6587 | -0.049 | -0.0847 | No |
| 51 | ADCY8 | adenylate cyclase 8 [Source:HGNC Symbol;Acc:HGNC:239] | 6646 | -0.053 | -0.0874 | No |
| 52 | GUCY1A2 | guanylate cyclase 1 soluble subunit alpha 2 [Source:HGNC Symbol;Acc:HGNC:4684] | 6731 | -0.058 | -0.0919 | No |
| 53 | ITPR1 | "inositol 1,4,5-trisphosphate receptor type 1 [Source:HGNC Symbol;Acc:HGNC:6180]" | 6752 | -0.059 | -0.0915 | No |
| 54 | NPR2 | natriuretic peptide receptor 2 [Source:HGNC Symbol;Acc:HGNC:7944] | 6771 | -0.061 | -0.0908 | No |
| 55 | PLA2G5 | phospholipase A2 group V [Source:HGNC Symbol;Acc:HGNC:9038] | 6772 | -0.061 | -0.0887 | No |
| 56 | MAPK1 | mitogen-activated protein kinase 1 [Source:HGNC Symbol;Acc:HGNC:6871] | 6853 | -0.068 | -0.0926 | No |
| 57 | PLCB2 | phospholipase C beta 2 [Source:HGNC Symbol;Acc:HGNC:9055] | 7052 | -0.084 | -0.1052 | No |
| 58 | PLCB3 | phospholipase C beta 3 [Source:HGNC Symbol;Acc:HGNC:9056] | 7118 | -0.090 | -0.1072 | No |
| 59 | PRKCH | protein kinase C eta [Source:HGNC Symbol;Acc:HGNC:9403] | 7141 | -0.092 | -0.1057 | No |
| 60 | RAMP2 | receptor activity modifying protein 2 [Source:HGNC Symbol;Acc:HGNC:9844] | 7247 | -0.099 | -0.1105 | No |
| 61 | GNA12 | G protein subunit alpha 12 [Source:HGNC Symbol;Acc:HGNC:4380] | 7294 | -0.102 | -0.1106 | No |
| 62 | CALD1 | caldesmon 1 [Source:HGNC Symbol;Acc:HGNC:1441] | 7359 | -0.107 | -0.1119 | No |
| 63 | PLA2G2E | phospholipase A2 group IIE [Source:HGNC Symbol;Acc:HGNC:13414] | 7486 | -0.117 | -0.1177 | No |
| 64 | GNAQ | G protein subunit alpha q [Source:HGNC Symbol;Acc:HGNC:4390] | 7518 | -0.120 | -0.1160 | No |
| 65 | ARAF | "A-Raf proto-oncogene, serine/threonine kinase [Source:HGNC Symbol;Acc:HGNC:646]" | 8092 | -0.163 | -0.1552 | No |
| 66 | BRAF | "B-Raf proto-oncogene, serine/threonine kinase [Source:HGNC Symbol;Acc:HGNC:1097]" | 8171 | -0.169 | -0.1554 | No |
| 67 | PLA2G1B | phospholipase A2 group IB [Source:HGNC Symbol;Acc:HGNC:9030] | 8232 | -0.173 | -0.1541 | No |
| 68 | ADCY3 | adenylate cyclase 3 [Source:HGNC Symbol;Acc:HGNC:234] | 8572 | -0.199 | -0.1738 | No |
| 69 | PRKCG | protein kinase C gamma [Source:HGNC Symbol;Acc:HGNC:9402] | 8582 | -0.200 | -0.1676 | No |
| 70 | RHOA | ras homolog family member A [Source:HGNC Symbol;Acc:HGNC:667] | 8601 | -0.201 | -0.1620 | No |
| 71 | CYP4A11 | cytochrome P450 family 4 subfamily A member 11 [Source:HGNC Symbol;Acc:HGNC:2642] | 8654 | -0.205 | -0.1590 | No |
| 72 | GNAS | GNAS complex locus [Source:HGNC Symbol;Acc:HGNC:4392] | 8929 | -0.226 | -0.1726 | No |
| 73 | MYL6 | myosin light chain 6 [Source:HGNC Symbol;Acc:HGNC:7587] | 9188 | -0.247 | -0.1843 | No |
| 74 | ADORA2B | adenosine A2b receptor [Source:HGNC Symbol;Acc:HGNC:264] | 9255 | -0.252 | -0.1808 | No |
| 75 | GUCY1A1 | guanylate cyclase 1 soluble subunit alpha 1 [Source:HGNC Symbol;Acc:HGNC:4685] | 9420 | -0.266 | -0.1844 | No |
| 76 | CALCRL | calcitonin receptor like receptor [Source:HGNC Symbol;Acc:HGNC:16709] | 9584 | -0.282 | -0.1874 | Yes |
| 77 | NPR1 | natriuretic peptide receptor 1 [Source:HGNC Symbol;Acc:HGNC:7943] | 9634 | -0.286 | -0.1814 | Yes |
| 78 | ADCY2 | adenylate cyclase 2 [Source:HGNC Symbol;Acc:HGNC:233] | 9715 | -0.293 | -0.1775 | Yes |
| 79 | CACNA1D | calcium voltage-gated channel subunit alpha1 D [Source:HGNC Symbol;Acc:HGNC:1391] | 9867 | -0.308 | -0.1787 | Yes |
| 80 | MAP2K2 | mitogen-activated protein kinase kinase 2 [Source:HGNC Symbol;Acc:HGNC:6842] | 9932 | -0.315 | -0.1728 | Yes |
| 81 | ADRA1D | adrenoceptor alpha 1D [Source:HGNC Symbol;Acc:HGNC:280] | 10031 | -0.325 | -0.1693 | Yes |
| 82 | ARHGEF11 | Rho guanine nucleotide exchange factor 11 [Source:HGNC Symbol;Acc:HGNC:14580] | 10056 | -0.326 | -0.1599 | Yes |
| 83 | CALM1 | calmodulin 1 [Source:HGNC Symbol;Acc:HGNC:1442] | 10136 | -0.334 | -0.1545 | Yes |
| 84 | PLA2G2A | phospholipase A2 group IIA [Source:HGNC Symbol;Acc:HGNC:9031] | 10152 | -0.336 | -0.1441 | Yes |
| 85 | MAP2K1 | mitogen-activated protein kinase kinase 1 [Source:HGNC Symbol;Acc:HGNC:6840] | 10197 | -0.340 | -0.1358 | Yes |
| 86 | ITPR3 | "inositol 1,4,5-trisphosphate receptor type 3 [Source:HGNC Symbol;Acc:HGNC:6182]" | 10390 | -0.360 | -0.1384 | Yes |
| 87 | PRKACG | protein kinase cAMP-activated catalytic subunit gamma [Source:HGNC Symbol;Acc:HGNC:9382] | 10466 | -0.370 | -0.1315 | Yes |
| 88 | CALM2 | calmodulin 2 [Source:HGNC Symbol;Acc:HGNC:1445] | 10579 | -0.382 | -0.1271 | Yes |
| 89 | PRKACA | protein kinase cAMP-activated catalytic subunit alpha [Source:HGNC Symbol;Acc:HGNC:9380] | 10611 | -0.384 | -0.1163 | Yes |
| 90 | PLA2G2F | phospholipase A2 group IIF [Source:HGNC Symbol;Acc:HGNC:30040] | 10628 | -0.386 | -0.1042 | Yes |
| 91 | ROCK2 | Rho associated coiled-coil containing protein kinase 2 [Source:HGNC Symbol;Acc:HGNC:10252] | 10648 | -0.388 | -0.0923 | Yes |
| 92 | PRKG1 | protein kinase cGMP-dependent 1 [Source:HGNC Symbol;Acc:HGNC:9414] | 10776 | -0.403 | -0.0884 | Yes |
| 93 | PLA2G6 | phospholipase A2 group VI [Source:HGNC Symbol;Acc:HGNC:9039] | 11128 | -0.446 | -0.1004 | Yes |
| 94 | PPP1R12A | protein phosphatase 1 regulatory subunit 12A [Source:HGNC Symbol;Acc:HGNC:7618] | 11259 | -0.465 | -0.0945 | Yes |
| 95 | PRKX | protein kinase X-linked [Source:HGNC Symbol;Acc:HGNC:9441] | 11634 | -0.522 | -0.1057 | Yes |
| 96 | PPP1CB | protein phosphatase 1 catalytic subunit beta [Source:HGNC Symbol;Acc:HGNC:9282] | 11737 | -0.539 | -0.0951 | Yes |
| 97 | ADCY6 | adenylate cyclase 6 [Source:HGNC Symbol;Acc:HGNC:237] | 12005 | -0.594 | -0.0954 | Yes |
| 98 | ROCK1 | Rho associated coiled-coil containing protein kinase 1 [Source:HGNC Symbol;Acc:HGNC:10251] | 12046 | -0.605 | -0.0777 | Yes |
| 99 | JMJD7-PLA2G4B | JMJD7-PLA2G4B readthrough [Source:HGNC Symbol;Acc:HGNC:34449] | 12076 | -0.614 | -0.0588 | Yes |
| 100 | GNA11 | G protein subunit alpha 11 [Source:HGNC Symbol;Acc:HGNC:4379] | 12375 | -0.700 | -0.0579 | Yes |
| 101 | ARHGEF1 | Rho guanine nucleotide exchange factor 1 [Source:HGNC Symbol;Acc:HGNC:681] | 12673 | -0.846 | -0.0520 | Yes |
| 102 | AVPR1A | arginine vasopressin receptor 1A [Source:HGNC Symbol;Acc:HGNC:895] | 12751 | -0.913 | -0.0265 | Yes |
| 103 | GNA13 | G protein subunit alpha 13 [Source:HGNC Symbol;Acc:HGNC:4381] | 12862 | -1.103 | 0.0030 | Yes |
Table: GSEA details [plain text format]

  

Fig 2: KEGG\_VASCULAR\_SMOOTH\_MUSCLE\_CONTRACTION      
 Blue-Pink O' Gram in the Space of the Analyzed GeneSet

  

Fig 3: KEGG\_VASCULAR\_SMOOTH\_MUSCLE\_CONTRACTION: Random ES distribution      
 Gene set null distribution of ES for **KEGG\_VASCULAR\_SMOOTH\_MUSCLE\_CONTRACTION**
